# Effect of Six-Minute Walk Test and Incremental Exercise on Inspiratory Capacity, Ventilatory Constraints, Breathlessness and Exercise Performance in Sedentary Male Smokers without Airway Obstruction

**DOI:** 10.3390/ijerph182312665

**Published:** 2021-12-01

**Authors:** Wassim Melliti, Rim Kammoun, Donies Masmoudi, Said Ahmaidi, Kaouthar Masmoudi, Fawaz Alassery, Habib Hamam, Mehdi Chlif

**Affiliations:** 1 Research Unit: Education, Motor Skills, Sport and Health (EM2S), UR15JS01, High Institute of Sport and Physical Education, University of Sfax, Sfax BP 3000, Tunisia; mallitiwassim@gmail.com; 2 Research Unit Respiratory Pathology in Southern Tunisia, Pulmonology Department CHU Hedi Chaker, Sfax 3000, Tunisia; 3 Physiology and Functional Exploration Service, University Hospital Habib Bourguiba, Sfax 3000, Tunisia; rimkammoun@yahoo.fr (R.K.); doniesmasmoudi@gmail.com (D.M.); Kaouthar.masmoudi.km@gmail.com (K.M.); 4 EA 3300 “APS and Motor Patterns: Adaptations-Rehabilitation”, Picardie Jules Verne University, 80025 Amiens, France; said.ahmaidi@gmail.com; 5 Department of Computer Engineering, College of Computers and Information Technology, Taif University, P.O. Box 11099, Taif 21944, Saudi Arabia; falasser@tu.edu.sa; 6 Department of Electrical Engineering, Faculty of Engineering, Moncton University, Moncton, NB E1A 3E9, Canada; Habib.Hamam@umoncton.ca; 7 National Center of Medicine and Science in Sports (NCMSS), Tunisian Research Laboratory Sports Performance Optimization, Ave Med Ali Akid, El Menzah, Tunis 263, Tunisia

**Keywords:** smoking, ventilatory efficiency, mechanical constraints, breathlessness, inspiratory capacity, incremental exercise

## Abstract

We investigated physiological responses and exercise capacity in sedentary young smokers during sub-maximal and maximal test and its impact on dyspnea and exercise intolerance. Fifty sedentary male smokers and non-smokers (age: 24 ± 1 years., weight: 71 ± 9 kg, height: 177.3 ± 4.8 cm, body mass index: 22.6 ± 2.5 kg/m^2^) underwent two visits with pulmonary function tests, breathing pattern, and inspiratory capacity measurement at rest and during sub-maximal and maximal exercise. Smokers show reduced exercise capacity during six minutes walk test (6-MWT) with decreased walked distance (*p* < 0.001) and inspiratory capacity (*p* < 0.05). During cardiopulmonary exercise test (CPET), smokers had higher minute ventilation VE for a given submaximal intensity (*p* < 0.05) and lower minute ventilation at maximal exercise (*p* < 0.001). End expiratory lung volume was significantly lower in sedentary smokers at rest (*p* < 0.05), at ventilatory threshold during exercise (*p* < 0.05), but not during peak exercise. End inspiratory lung volume was significantly lower in smokers at rest (*p* < 0.05) and ventilatory threshold (*p* < 0.05). Cigarette smoking alters lung function during submaximal and maximal exercise. This alteration is manifested by the development of dynamic hyperinflation contributing to exercise capacity limitation.

## 1. Introduction

Smoking is undoubtedly a major cause of illness and premature death, as well as a source of discomfort and possibly poorer quality of life in smokers or those exposed to tobacco smoke [1]. Smokers without bronchial obstruction but who report persistent respiratory symptoms (cough, sputum, or dyspnea) have a higher risk of all-cause mortality and an accelerated decline in lung function compared to healthy subjects [2]. This subpopulation was previously designated by the gold obstructive lung diseases (GOLD) at stage 0 of chronic obstructive pulmonary disease at risk for chronic obstructive pulmonary disease (COPD) [3]. Recent studies have confirmed that former and current smokers without chronic obstructive pulmonary disease (COPD) have a lower perceived quality of life, greater respiratory symptoms (including exertional dyspnea), and poor exercise tolerance than healthy non-smoker [4].

Tests of maximum ability and endurance capacity are two methods for measuring the performance of whole-body exercise. Most physicians assess their patients with questions related to respiratory function, such as walking [5]. This is perhaps one cause why the 6-min walk (6MWD) test has become a common alternative to the formalized cardiopulmonary training test. The 6MWD is representative of daily life activities and a reliable method for assessing the functional status of chronic heart and lung diseases patients [6]. Since exercise intolerance is a common complaint, cardiopulmonary exercise testing (CPET) may provide additional information. CPET is becoming more common as a clinical diagnostic method for evaluating exercise intolerance and exertional effects, as well as objectively determining functional ability and disability [7]. In the face of increased ventilation requirements during exercise in research and clinical environments, CPET is especially suitable for understanding factors that could limit ventilation.

The evaluation of respiratory function by measuring pulmonary volumes, inspiratory and expiratory flow rates, and various gas exchange parameters gives indirect and useful information concerning the functioning of the ventilatory function during exercise [8]. Ventilatory limitation and exercise tolerance and exertional dyspnea were strongly investigated in heavy smokers or with associated diseases. However, in sedentary young smokers, this relation is still not elucidated, warranting further study. Respiratory response in sedentary smokers may be a limiting factor in exercise. The specific adaptations of the respiratory system could notably result in the appearance of mechanical and metabolic ventilatory constraints during a maximal or even sub-maximal exercise, reflecting the balance between ventilatory demand and ventilatory capacity [9]. Ventilatory limitations were traditionally determined by the measurements of how close minute ventilation (VE) reaches the maximum voluntary ventilation (MVV) during maximal exercise. The ventilatory reserve (VE/MVV) ratio is relatively insensitive and gives little information on the specific reasons for ventilatory constraint and does not give insight into respiratory strategy or the level of restrictions on expiratory or inspiratory flow [10]. Understandably, there is considerable controversy surrounding the assessment of the ventilation reserve in part due to the lack of a definitive method for measuring the ventilation capacity. However, emerging technologies (such as specific exercise tidal flow volume loops regarding maximum flow-volume loops) have provided valuable additional insight into how mechanical constraints limit exercise [8]. The flow-volume curve is one of the non-invasive techniques used to quantify the ventilatory constraints induced by exercise and to give help during the diagnosis and the localization of airway obstruction [8,10,11]. Dynamic hyperinflation (DH) refers to gradual inflation in lung volumes during exercise in response to a rise in VE. The existence of DH has been verified in many studies of COPD, in which DH has been found to contribute significantly to increased dyspnea and exercise limitations.

The major objective of this study is to investigate the mechanical ventilatory responses in sedentary young smokers using inspiratory capacity determination throughout the maximal and sub-maximal exercise. We hypothesized that DH during exercise is associated with increased dyspnea and exercise limitation.

## 2. Materials and Methods

### 2.1. Subjects

Fifty male students were recruited for the study. Participants were chosen according to a systematic random process. All participants were sedentary aged between 23 and 25 years old. Participants’ lifestyle was interrogated to confirm their sedentary behavior. According to the Sedentary Behavior Research Network, the latter is defined as any waking behavior such as sitting or leaning with an energy expenditure of less than 1.5 metabolic equivalents units (METs). (One MET is the energy cost of resting quietly, often defined in terms of oxygen uptake as 3.5 mLO_2_·kg^−1^·min^−1^) [12].

The procedures of the study were approved by the hospital ethics committee and complied with the ethical standards of the Helsinki Declaration of 1975.

The nature and purpose of the research were explained to the participants before giving their verbal acceptance and signed consent. A pack-year is a clinical quantification of cigarette smoking used to define tobacco exposure. It is calculated by multiplying the number of packs of cigarettes smoked per day by the number of years the person has smoked. Smokers should have a smoking history of at least 3 pack years. This is used to assess the risk of developing pathologies related to tobacco use [13]. Participants underwent regular medical monitoring and their clinical or functional status did not change during the two months preceding the study. No subject had a history of asthma, active heart disease, musculoskeletal disorders, peripheral vascular disease, or other disabling conditions that interfere with testing. Subjects not meeting these guidelines were excluded.

### 2.2. Protocol

Participants completed two visits. The first visit involved medical history investigation, pulmonary function tests, six minutes walk test (6-MWT), inspiratory capacity (IC), and maximal inspiratory pressure (MIP) measurements during rest (pre-exercise) and immediately after finishing the exercise (post-exercise). To avoid effects of time of day all tests were conducted at the same time (10 a.m.). The second visit took place 3 weeks after the first one. The second visit included pulmonary function test followed by an incremental cycle CPET with inspiratory capacity measurement and maximal inspiratory pressure measurements before and immediately after exercise. Participants were informed to avoid: (i) ingestion of alcohol, food, and caffeine for at least 2 h before exercise testing, (ii) smoking for at least 12 h before exercise testing and refraining from strenuous activity for at least 12 h. Subjects were not tested if they had had respiratory tract infections within 3 weeks.

### 2.3. Body Composition

Measurements of weight and height preceded the spirometry. Height was measured with a Harpenden stadiometer 602VR to the last complete 0.1 cm and body composition was estimated using a multi-frequency bioelectrical impedance analyzer (TBF-410GS, Tanita Co., Tokyo, Japan). This is a method validated against the reference methods [14]. The collected parameters were weight (Wt) and body mass index (BMI).

### 2.4. Pulmonary Function Tests

Spirometry was used in all subjects participating in this study, and parameters determined were: forced expiratory volume second (FEV1); forced vital capacity (FVC); peak expiratory flow (PEF); and FEV1/FVC% ratio. The subjects performed at least three measurements; the criteria of validity and reproducibility are those of the ATS/ERS [15]. Global lung function initiative (GLI) predicted values were used [16].

In addition, Static lung volumes and maximum inspiratory capacity were determined in a constant-volume whole body plethysmograph (Body Box 5500 Series Morgan Scientific Inc. 151 Essex St. Ste 8 Haverhill, MA, USA) following recommended techniques [14]. The predictive values for lung function parameters were derived from those published by Clausen et al. [17].

### 2.5. Maximal Inspiratory Pressure 

Maximal inspiratory pressure (MIP) was measured at the Functional Residual Capacity (FRC) on seated subjects at rest and immediately after the exercise test using the technique of Black and Hyatt [18]. The maximal inspiratory pressure (MIP) was measured with a manometer connected to a mouthpiece (Micro RPM, Care Fusion, UK) according to international guidelines [19]; subjects had no previous experience of these maneuvers. Therefore, great care was taken to explain the procedures. Subjects were asked to perform a maximal inspiratory effort against an occluded airway and to maintain this for at least one second; the maneuver was repeated for a minimum of three attempts and reproducibility had to be within <5%. The best score was kept for analysis.

### 2.6. Six Minutes Walk Test (6-MWT)

The 6-MWD was performed indoors along a flat, straight, 30-m walking course and measured using the better of two tests separated by ≥30 min according to the American thoracic society guidelines [5]. Before and after the 6MWT, the following variables were recorded: heart rate (HR); oxygen saturation using pulse oximetry (SpO_2_); and rate of perceived breathlessness using the Borg scale (0–10). Participants were encouraged every minute during the test. They were allowed to stop and rest during the test but were instructed to resume walking as soon as they felt able to do so. At the end of the test, the 6-min walked distance was recorded. A medical doctor conducted the IC calculation after thoroughly explaining the technique to each subject. Inspiratory capacity (IC) was measured with the subject in the standing position at rest and immediately after the 6MWT. IC maneuver instructions were given to all subjects. The subjects were advised to inspire to total lung capacity (TLC) and then return to regular breathing after four to six consistent end-expiratory levels. One IC was registered (from at least three acceptable trials, the two largest IC measurements had to agree within 5% or 60 mL before 6MWT, and only one measure at the end). Dynamic hyperinflation was defined as a decrease of >150 mL or 10% in IC at the end of exercise compared with resting levels. Inspiratory capacity was measured according to international guidelines [20].

### 2.7. Cardiopulmonary Exercise Testing (CPET)

Exercise testing was performed on a cycle ergometer (Ergometrics 800S, SensorMedics, Anaheim, CA, USA). After 3 min of baseline measurements, the subjects started cycling. Thereafter, the work rate increased incrementally in 1-min intervals until a symptom-limited endpoint was reached. The individualized exercise test protocol used in our laboratory usually results in a maximal oxygen uptake test duration of 8–12 min, meeting standard exercise testing recommendations [7]. Exercise variables were measured and averaged over the last 30 s of 1-min increments and at peak exercise.

All subjects were encouraged to exercise until exhaustion of the point at which they felt unable to continue. Test termination criteria included: (i) volitional exhaustion, (ii) leveling off oxygen uptake (VO_2_), (iii) pedal rate note maintained at 50 rpm, (iv) respiratory exchange ratio (RER) greater than 1.15, (v) maximal heart rate (HRmax) ≥ 90% of theoretical HR max. Peak VO_2_ was defined as the highest VO_2_ that could be sustained for at least 30 s during the last stage of exercise [21]. At the end of the test, each subject had a 2-min active recovery and a 3-min passive recovery.

Both inspiratory and expiratory airflow were obtained from the calibrated mass flow sensor (Vmax 29 Metabolic Measurement System, Sensor Medics, Anaheim, CA, USA) and electronically integrated to obtain volume measurements. Measurements of oxygen consumption (VO_2_) and carbon dioxide production (VCO_2_) were made with the use of a computerized custom gas exchange. Ventilatory threshold (VTH) was determined as described previously [22,23]. Three validated methods were used concurrently to determine VTH from incremental exercise test data: (1) ventilatory equivalent method (VE/VO_2_ method) [23], (2) excess carbon dioxide method (PETCO_2_) [22], and (3) modified V slope method [24]. This point was measured in a double-blind design, according to the best agreement between two independent observers. In case of disagreement (i.e., more than 10% difference between the two observers), a third investigator was asked to assess the thresholds. The value retained was the average of the values in closer agreement. The ventilatory threshold was designated as the work rate that was most congruent among the different threshold determination methods. Electrocardiography and pulse oximetry were carried out continuously using finger sensor, and blood pressure was taken by auscultation at rest, at the end of each stage of exercise, at peak exercise, and during recovery from exercise. oxygen saturation (SpO_2_) was measured noninvasively with a pulse oximeter at the finger to detect exercise-induced hypoxemia, which was defined as a drop in SpO_2_ of 3–4% between rest and the end of the exercise. SpO_2_ % was designated as arterial oxygen desaturation as indicated, by pulse oximetry (SpO_2_ rest minus SpO_2_ maximal exercise). The modified Borg scale was used to record rating of perceived breathlessness (RPB) and perceived exertion at peak exercise. To avoid possible effects of performing IC maneuvers on dyspnea intensity, IC maneuvers were always performed after subjects completed symptom intensity ratings.

### 2.8. Flow-Volume Measurements

To quantify exercise-induced ventilatory constraints we used the exercise volume-flow curve. The advantage of this technique is to determine the degree of expiratory ventilatory limitations, the respiratory strategies adopted, the imposed elastic load, inspiratory reserves, and an estimate of ventilatory capacity.

The principle of this method is to calculate inspiratory capacity (IC). Therefore, inspiratory capacity was measured at rest immediately after 6-MWT, before CPET, and during the last 30 s of each level of maximal exercise to determine the placement of the volume flow curve at exercise (ext FVL) versus the baseline flow-volume curve (MFVL) [8]. Literature shows that IC maneuvers do not interfere with the main cardiorespiratory functional parameters used for the interpretation of the cardiopulmonary parameters during incremental exercise [25]. Assuming that the TLC remained constant during exercise, IC deviations of at least 10% from baseline were used to determine dynamic hyperinflation.

The end-expiratory lung volume (EELV) was estimated from the inspiratory capacity measurements (EELV = TLC − IC) while the end-inspiratory lung volume was calculated from the following equation: EILV = VT + EELV. This assumes that TLC does not change significantly with body position [26] or exercise [27]. The best pre-and post-exercise of the MFVL were plotted and placed relative to TLC to compare potential changes in inspiratory and expiratory flow and volume. The MFVL with the largest expiratory and inspiratory envelope was used as the reference flow-volume curve.

### 2.9. Data Analysis

Values are presented as the mean ± standard deviation (SD). An a priori power analysis was conducted using G*Power3 [28] to test the difference between two independent group means using a two-tailed test, a medium effect size (d = 0.50), and an alpha of 0.05. Results showed that a total sample of 50 participants with 25 smokers and 25 nonsmokers subjects was required to achieve a power of 0.80. Cohen’s d effect sizes (d) were classified as small (0.20), moderate (0.50), and large (0.80) and were calculated from data on the means differences, the number of subjects, and the mean pooled standard deviations (SD) [29]. Differences between the two groups were compared using an unpaired Student’s test or the Mann-Whitney test when both normality (Kolmogorov-Smirnov) and equality of the variance (Levene median test) test failed.

The maximal inspiratory pressure data were compared before and after six-minute walking test and incremental exercise to rest condition using a paired Student’s *t*-test or a Wilcoxon ranks test in the absence of normality.

Analyses were carried out using SPSS software (IBM Corp. Released 2016. IBM SPSS Statistics for Windows, Version 25.0, Armonk, NY, USA). Statistical analysis was performed applying a significance level of 0.05.

## 3. Results

### 3.1. Subjects

Anthropometric characteristics of sedentary young smokers and sedentary non-smokers are presented in Table 1. There were no significant differences between the groups in age, weight, height, and BMI.

### 3.2. Pulmonary Function

Pulmonary function and exercise data of the two groups, sedentary smokers and sedentary non-smokers, are presented in Table 1. All participants had normal spirometry parameters. However, sedentary smokers had a lower FEV1/FVC ratio (*p* < 0.05, d = 0.87) compared to non-smokers.

### 3.3. Six Minutes Walk Test (6-MWT)

The 6-MWT values are resumed in Table 2. Sedentary young smokers presented a significantly lower walked distance (*p* < 0.01, d = 1.18) and oxygen saturation (*p* < 0.001, d = 1.19) compared to non-smokers. Heart rate (HR) was not significantly different between sedentary non-smokers and smokers, at rest and after 6-MWT. Reduction of inspiratory capacity (IC) measured immediately after 6-MWT was statistically more important in sedentary young smokers compared to non-smokers (*p* < 0.05, d = 0.6).

### 3.4. Cardiopulmonary Exercise Testing (CPET)

All of the subjects reached maximal exercise during CPET. The values of the cardio-metabolic parameters obtained at maximal exercise are listed in Table 3. At maximal exercise, sedentary non-smokers achieved a higher workload (*p* < 0.05, d = 3.16) and working time (*p* < 0.05, d = 2) and higher oxygen uptake than sedentary smokers (*p* < 0.05, d = 1.8). The oxygen pulse (VO_2_/HR) was higher in non-smokers at maximal exercise (*p* < 0.01, d = 0.63). Arterial oxygen desaturation was significantly lower in sedentary young smokers than non-smokers (*p* < 0.05, d = 0.61). Rates of perceived exertion (RPE) and breathlessness (RPB) values were not significantly different between the two groups.

### 3.5. Ventilatory Responses to Exercise

**Figure 1:**
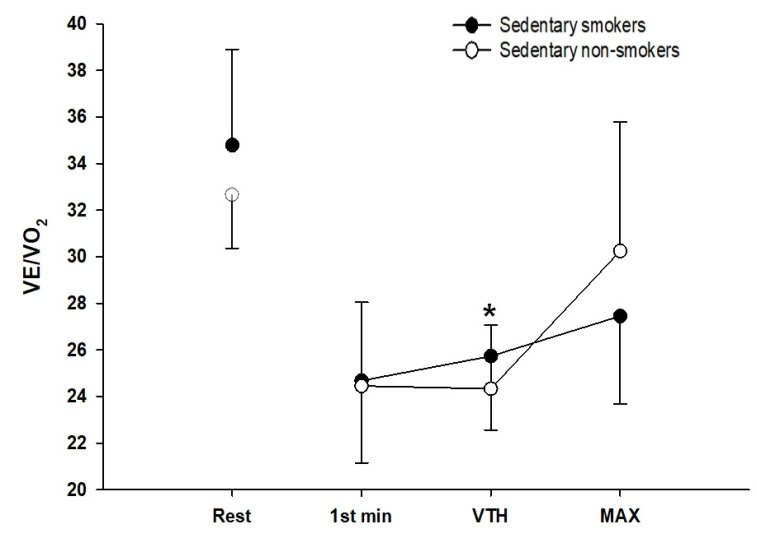
Ventilation Equivalents in O_2_ (VE/VO_2_) in sedentary smokers (black circles) (*n* = 25) and sedentary non-smokers (white circles) (*n* = 25). *t*-test for independent samples is used for analysis. Data represented at rest, first minute of exercise (1st min), ventilatory threshold (VTH), and maximal exercise (Max), respectively; * *p* < 0.05.

**Figure 2:**
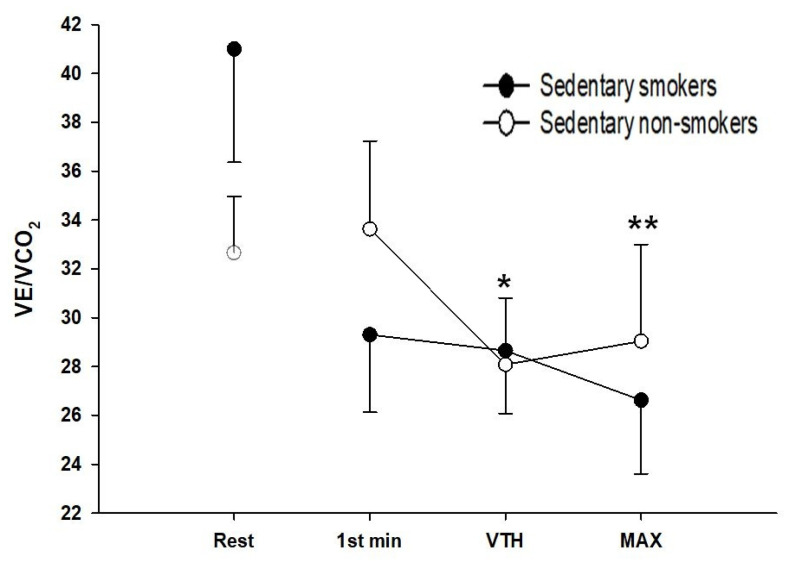
Ventilation equivalents in CO_2_ (VE/CO_2_) in sedentary smokers (black circles) (*n* = 25) and sedentary non-smokers (white circles) (*n* = 25). *t*-test for independent samples is used for analysis. Data represented at rest, first minute of exercise, ventilatory threshold, and maximal exercise, respectively; Sedentary smokers vs. sedentary non-smokers: * *p* < 0.05; ** *p* < 0.01.

The evolution of ventilatory equivalents in O_2_ (VE/VO_2_) and CO_2_ (VE/VCO_2_) during exercise is shown in Figure 1 and Figure 2.

**Figure 3:**
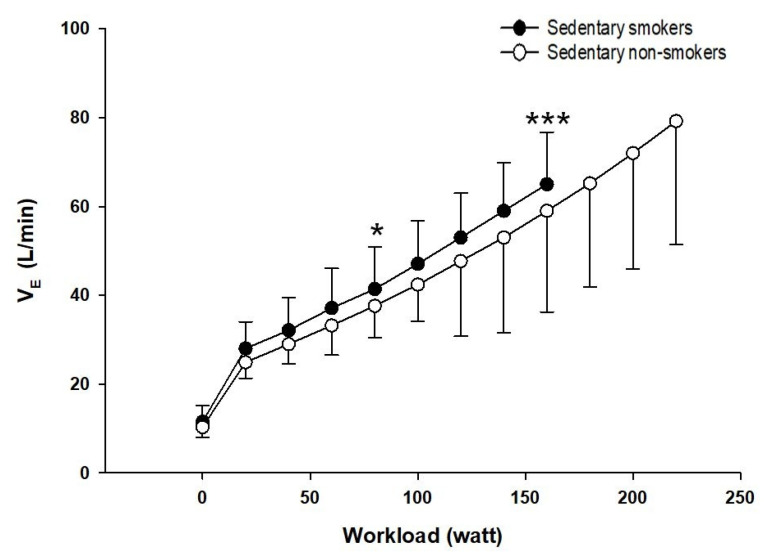
Minute ventilation (VE)at comparable (50, 100, 150 and 200 W) and at maximal workloads in and sedentary non-smokers (white circles) (*n* = 25). *t*-test for independent samples is used for analysis. Sedentary smokers vs. sedentary non-smokers: * *p* < 0.05; *** *p* < 0.001.

The two groups show no significant difference at rest and the first level of exercise. At the ventilatory threshold, VE/VO_2_ was significantly lower (*p* < 0.05, d = 0.46) while VE/VCO_2_ was higher in sedentary young smokers compared to controls (*p* < 0.05, d = 0.21). At maximal exercise, VE/VCO_2_ was significantly lower in sedentary young smokers than non-smokers (*p* < 0.01, d = 0.68). Minute ventilation (VE) is plotted against the work rate (Figure 3). There were no significant differences observed in VE at rest and the first level of exercise. However, sedentary young smokers had higher ventilation at the ventilatory threshold, but lower at maximal exercise than sedentary non-smokers (*p* < 0.001, d = 0.66).

### 3.6. Breathing Mechanics

**Figure 4:**
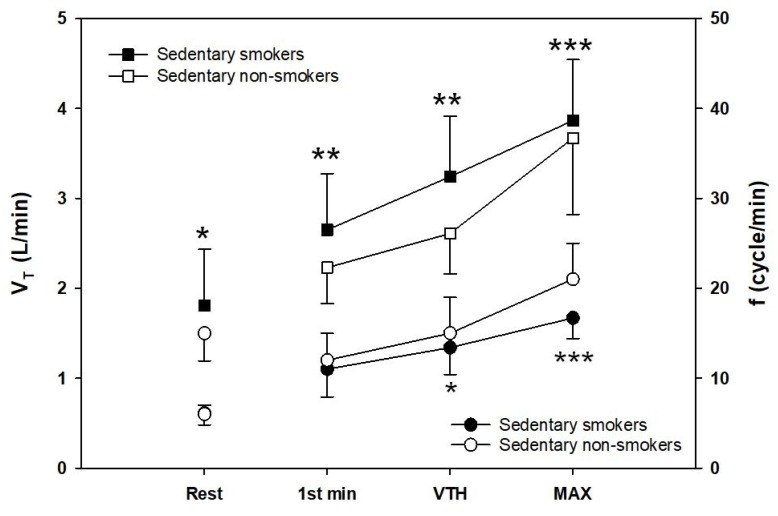
Tidal volume V_T_ (squares) and breathing frequency f (circles) plotted against minute ventilation. *t*-test for independent samples is used for analysis. Data represented at rest, first minute of exercise, ventilatory threshold, and maximal exercise, respectively; Sedentary smokers vs. sedentary non-smokers: * *p* < 0.05; ** *p* < 0.01; *** *p* < 0.001.

Tidal volume (VT) and breathing frequency (f) are plotted against VE at rest, the first level of exercise, ventilatory threshold, and peak exercise (Figure 4). No differences in tidal volume were observed between groups at rest and the first level of exercise. Sedentary young smokers had a lower tidal volume than control at the ventilatory threshold (*p* < 0.05, d = 0.44) and at peak exercise (*p* < 0.001, d = 1.30). Breathing frequency (f) was significantly increased by smoking. Sedentary young smokers had a greater (f) relative to sedentary non-smokers at rest (*p* < 0.05, d = 0.6), the first level of exercise, and at the ventilatory threshold (*p* < 0.01, d = 1.07). At peak exercise, (f) was significantly higher in sedentary young smokers than controls (*p* < 0.001, d = 0.25). End of expiratory volume (EELV (% TLC)) was significantly lower in smokers at rest (*p* < 0.05, d = 0.82) and at VTH (*p* < 0.05, d = 1.13), but not during peak exercise. During heavy to peak exercise, sedentary young smokers increased EELV to levels above resting values, whereas sedentary non-smokers continued to decrease EELV.

EILV (% TLC) was significantly lower in sedentary young smokers at rest and at VT (*p* < 0.05, d = 0.82) but not during peak exercise. EELV (%TLC) and EILV (%TLC) kinetics are shown in Table 4.

### 3.7. Maximal Inspiratory Pressure

**Figure 5:**
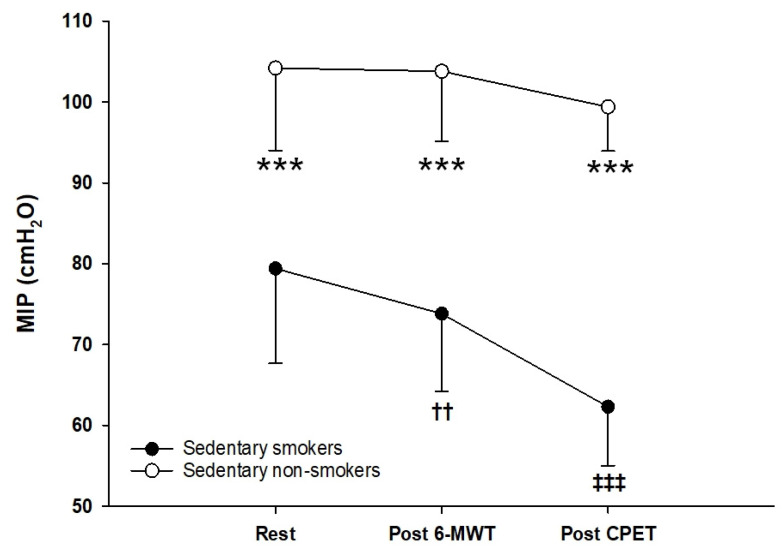
Mean maximal inspiratory pressure (MIP) at rest, after sub-maximal exercise (6-MWT) and maximal exercise (CPET) in sedentary smokers (black circles) (*n* = 25) and sedentary non-smokers (white circles) (*n* = 25). *t*-test for independent samples is used for analysis; *** *p* < 0.001; Rest, Post-6-MWT compared in sedentary smokers versus non-smokers. *t*-test for dependent samples is used for analysis of Post-6-MWT and post CPET compared to rest in sedentary smokers and non-smokers: ^††^ *p* < 0.01; ^‡‡‡^ *p* < 0.001.

Maximal inspiratory pressure was significantly lower in sedentary young smokers compared to sedentary non-smokers (*p* < 0.001, d = 2.2). Results are summarized in Figure 5. Maximal inspiratory pressure values were statistically lower after 6-MWT (*p* < 0.01, d = 0.51) and CPET (*p* < 0.001, d = 1.68) in smokers. However, no differences were seen between the pre- and post-exercises maximal inspiratory pressure (MIP) in non-smokers.

## 4. Discussion

Our present findings indicate that during submaximal exercise such as 6MWT, smokers with no airway obstruction experience dynamic hyperinflation which can be determined by measuring IC. After CPET, this phenomenon is more pronounced. Nonetheless, the disparity in dynamic hyperinflation (DH) on both measures, which is higher after the gradual CPET, can be due to different exercise intensities manifested by variations in oxygen uptake, ventilatory efficiency, mechanical ventilatory constraints, oxygen desaturation, and breathlessness.

The relationship between smoking and pulmonary function has been widely studied [30,31,32]. Allinson et al. [33] reported that smoking decreased the FVC and FEV1. Guerra et al. [34] noted that the FEV1 and FVC could be considered as predictors of good or bad lung function. In our study, smokers had normal FVC and FEV1 values. However, FEV1/FVC ratio was significantly lower compared to non-smokers which may be a predictor of obstructive lung disease.

The 6-MWT evaluates the global responses of all vital systems. Although it does not evaluate the causes of exercise limitation, it provides information that may be a better index of participant’s ability to perform simple daily activities. Our results show that smokers have reduced exercise capacity as indicated, by a decreased distance walked compared to nonsmokers. The latter may reflect lower maximal oxygen consumption in smokers compared to non-smokers. This finding supports previous research results reported [35,36].

Inspiratory capacity (IC) was measured before and immediately at the end of the walking test. Our results show a drop of ≥200 mL in IC after 6-MWT in sedentary smokers compared to baseline values. According to the literature, this decrease results in dynamic hyperinflation (DH) associated with impaired exercise performance [37,38]. Dynamic hyperinflation leads to the following negative effects: (1) DH results in functional inspiratory muscle weakness by maximally shortening the muscle fibers in the diaphragm and reducing in consequence respiratory muscle strength [39]. In our study, maximal inspiratory pressure (MIP) was significantly lower in sedentary smokers compared to non-smokers at rest. Moreover, MIP values were significantly lower compared to rest in smokers. (2) DH increases the work and oxygen cost of breathing with a sudden increase in the elastic and threshold loads on the inspiratory muscles [40]. (3) DH reduces the ability of tidal volume to expand appropriately during exercise, leading to early mechanical limitation of ventilation [41]. All those factors lead to dyspnea and exercise limitation.

The cardiopulmonary exercise test shows that there was no difference between the two groups in minute ventilation (VE) at rest and the beginning of the exercise. However, at maximal exercise VE was higher in non-smokers than smokers. Reduced maximal VE in smokers may be related to the chronic effects of smoking on pulmonary function. Our result was following previous studies demonstrating that VE was greater during heavy work [42,43]. Moreover, smokers had a higher VE for a given submaximal exercise intensity compared to non-smokers with a lower VO_2_ leading to the elevation in VE/VO_2_. The latter must result from an increase in alveolar dead space, favoring units with higher ventilation relative to perfusion in the ventilation/perfusion distribution pattern. Our results have shown that smoking reduces the duration of the exercise and the time of occurrence of the first ventilatory threshold during maximal exercise. The early onset of shortness of breath is a factor of exercise intolerance that influenced the respiratory model adopted by sedentary smokers. At rest, the respiratory strategy was similar between both groups. VE increases linearly with increasing the intensity of exercise with a much larger contribution of VT than (f) in both groups. However, after the ventilatory threshold and at maximum exercise, non-smokers continued with the same strategy whereas, in smokers, we observe rapid and shallow breathing. The latter has a reduced VT. Therefore, to compensate for this fall they increase their respiratory frequency to have optimal ventilation. Rapid and shallow breathing can reduce fatigue of the respiratory muscles and maximum inspiratory muscle effort, and therefore optimize the O_2_ cost of breathing, because, for a given VE the combination of smaller VT and higher breathing frequency (f) is most efficient by reducing the loading related increased elastic forces against which smokers needed to breathe and increase the endurance of the inspiratory muscles. This strategy is advantageous initially but in the long term becomes ineffective as dead space increases and the O_2_ cost of breathing increases with increasing (f). Ventilatory efficiency is reflected by the ventilatory equivalent. In our study, ventilation and the ratio of ventilation to CO_2_ production (VE/VCO_2_) were elevated in sedentary young smokers at the ventilatory threshold and maximal exercise, proving this inefficiency. These suggest that their neuro-humoral ventilatory control mechanisms are intact. Potential mechanisms for this inefficient ventilation include both an increased physiological dead space and lower partial pressure of carbon dioxide (PaCO_2_) [44]. Our observation of increased peak VE/VCO_2_ ratio is consistent with the previous reports [45].

Mechanical constraints appear in sedentary smokers following this strategy. To quantify those exercise-induced ventilatory constraints we used the exercise flow volume loops method to measure the end of expiration lung volume (EELV) and end of inspiration lung volume (EILV). Our results show a decrease in resting EELV in sedentary smokers. This decrease probably reflects the decrease in pulmonary and thoracic compliance [46]. Similarly, this reduction seems to influence the regulation of the EELV during intensive exercise. It may be beneficial to keep the end-expiratory lung volume low, to optimize the VT and the length and strength of the inspiratory muscles. However, our results show that at maximum effort, members of the two groups further increased their EELV. In smokers, the EELV exceeds the basal values inducing an installation of dynamic hyperinflation. This decreases diaphragmatic efficiency and increases respiratory work. As the ventilatory demand increases and the subject increase his EELV to avoid the limitation of the expiratory flow and to take advantage of the maximum expiratory airflows available, the pulmonary volume of end of inspiration EILV also increases to maintain VT. The increase in EELV was coupled with an increase in EILV approaching TLC resulting in an excessive work of the inspiratory muscles. MIP was measured immediately after CPET, our results showed a decrease of nearly 20% in MIP values compared to rest in smokers. After incremental exercise, reduction in inspiratory strength might contribute to impairment in ventilatory capacity and dyspnea commonly observed in smokers. Moreover, this may indicate a reduced efficiency of the inspiratory muscle, which may lead to a greater risk of inspiratory muscle weakness. Furthermore, decreased respiratory muscle strength could reflect a generalized skeletal muscle weakness as mentioned in other populations [47].

Despite interesting results, some limitations must be pointed out. The number of participants recruited was relatively small, and the subjects of this study were all male. As such, additional researchers with a larger number of participants are required to clarify the significance of changes in young smokers compared to non-smokers. Participants were classified as light smokers; however, other important parameters such as smoking intensity and secondhand smoke were not taken into consideration which may affect the results. Moreover, the maximal inspiratory pressure test is volitional and requires full cooperation. Accordingly, a low result may sometimes owe to a lack of motivation and does not necessarily indicate reduced respiratory muscle strength. For this, using phrenic nerve stimulation in future studies is recommended.

## 5. Conclusions

Our study has been able to detect the development of DH in smokers during submaximal exercise such as the 6MWT and more pronouncedly during incremental CPET. IC Measurements were taken during exercise supplement standard measurements of ventilatory limitation and may provide further insight into exercise intolerance in smokers with preserved spirometry.

## Figures and Tables

**Table 1:** Baseline anthropometric characteristics and pulmonary function tests values in sedentary smokers and non-smokers.

	Sedentary Smokers (*n* = 25)	Sedentary Non-Smokers (*n* = 25)
Age, yr	24 ± 1	24 ± 2
Ht, cm	176.3 ± 6.8	178.3 ± 3
Wt, kg	70.5 ± 9.9	71.6 ± 8.5
BMI, kg/m^2^	22.8 ± 3.1	22.4 ± 2.2
BF (%)	14.48 ± 6.55	13.52 ± 5.06
Pack years	4 ± 1	0
FEV1 (L)	4.2 ± 0.7	4.5 ± 0.6
FEV1 (% predicted)	93.7 ± 13.7	97.5 ± 9.2
FVC (L)	5.2 ± 0.7	5.3 ± 0.8
FEV1/FVC (%)	79.6 ± 4.5 *	83.9 ± 5.5
PEF (L/sec)	6.7 ± 2.3 **	9.8 ± 2
PEF (% predicted)	63.6 ± 12.5 *	71.6 ± 5.04
TLC (L)	7.2 ± 1.1	7.8 ± 0.6
TLC (% predicted)	102.8 ± 12.3 *	107.6 ± 6.8
FRC (L)	4.2 ± 0.9	4.4 ± 0.6
FRC (% predicted)	129.8 ± 22.4	132.2 ± 16.4
RV (L)	2 ± 0.7	2.8 ± 0.4
RV (% predicted)	124.4 ± 38.6	152.2 ± 27.5

*t*-test for independent samples is used for analysis. Values are mean ± SD; yr: years, Ht: height, Wt: weight, BMI: body mass index; BF: body fat percent; FEV1: forced expiratory volume in 1 s; FEV1/FVC: forced expiratory volume in 1 sec to forced vital capacity ratio; FRC: functional residual capacity; RV: residual volume; FVC: forced vital capacity; PEF: peak expiratory flow; TLC: total lung capacity; Sedentary Smokers vs. Sedentary Non-Smokers: * *p* < 0.05; ** *p* < 0.01.

**Table 2:** Physiological response to the 6-MWT in sedentary smokers and non-smokers.

	Sedentary Smokers (*n* = 25)	Sedentary Non-Smokers (*n* = 25)
Distance (m)	584.8 ± 31.7 **	636.1 ± 52.2
HR baseline (beats/min)	72 ± 9	70 ± 8
HR peak (beats/min)	115 ± 14	104 ± 11
SBP. DBP baseline (mmHg)	112.73 ± 5.5	112.74 ± 7.2
SBP. DBP peak (mmHg)	113.76 ± 8.5	112.79 ± 9.7
SpO_2_ baseline (%)	98 ± 1	98 ± 1
SpO_2_ peak (%)	95 ± 1 ***	97 ± 1
Δ SpO_2_ (%)	−1.2 ± 1.2	−1.8 ± 1.7
ΔMIP (cmH2O)	−5.6 ± 2.13	−0.4 ± 1.52
ΔIC from rest, (L)	−0.2 ± 0.3 *	−0.1 ± 0.2
ΔIC (%)	6.1 ± 9	1.2 ± 5.3
Dyspnea baseline (Borg)	0	0
Dyspnea peak (Borg)	1	1

*t*-test for independent samples is used for analysis. Values are mean ± SD; SBP: systolic blood pressure, DBP: diastolic blood pressure HR: heart rate, IC: inspiratory capacity, MIP: maximal inspiratory pressure, SpO_2_: oxygen saturation; Δ: difference between basal and maximal exercise, Sedentary Smokers vs. Sedentary Non-Smokers: * *p* < 0.05; ** *p* < 0.01; *** *p* < 0.001.

**Table 3:** Cardiopulmonary exercise testing parameters at maximal exercise.

	Sedentary Smokers (*n* = 25)	Sedentary Non-Smokers (*n* = 25)
Duration (min)	8 ± 1 *	10 ± 1
Work Rate (Watt)	160 ± 20 *	210 ± 10
VO_2_ (L/min)	2.2 ± 0.3	2.4 ± 0.5
VO_2_ (mL/Kg/min)	35.2 ± 5 *	42.3 ± 2.5
HR (beats/min)	172 ± 18	181 ± 7
HR (%predicted)	89 ± 8	91 ± 5
VO_2_/HR (mL/beats/min)	13.4 ± 2.1 **	15.1 ± 3.1
RER	1 ± 0.15	1 ± 0.1
RPE (6–20)	18 ± 2	18 ± 1
RPB (0–10)	9 ± 2	6 ± 3
∆ SpO_2_	−3 ± 2 *	−2 ± 1

*t*-test for independent samples is used for analysis. Values are mean ± SD; HR: heart rate, RER: respiratory exchange ratio, RPB: rating of perceived breathlessness, RPE: rating of perceived exertion, SpO_2_: oxygen saturation, VO_2_: oxygen uptake, VO_2_/HR: oxygen pulse, ∆ SpO_2_: % arterial oxygen desaturation as indicated by pulse oximetry (SpO_2_ rest − SpO_2_ maximal exercise); Sedentary Smokers vs. Sedentary Non-Smokers: * *p* < 0.05; ** *p* < 0.01.

**Table 4:** End of Expiratory and Inspiratory lung volumes as percentage of Total Lung Capacity (TLC) in sedentary smokers and controls during CPET.

	EELV/TLC (%)			EILV/TLC (%)		
	Rest	VTH	Max	Rest	VTH	Max
Sedentary Smokers (*n* = 25)	59.3 ± 6.5 *	57 ± 3.4 *	61.8 ± 6.8	68 ± 6.9 *	73 ± 0.2 *	91 ± 5.9
Sedentary Non-Smokers (*n* = 25)	64.2 ± 4.9	62 ± 5.1	61.9 ± 6.6	72.2 ± 5.9	77 ± 6.3	87.4 ± 4.8

*t*-test for independent samples is used for analysis. Values are mean ± SD; EELV: end expiratory lung volume, EILV: end inspiratory lung volume, TLC: total lung capacity; Sedentary Smokers vs. Sedentary Non-Smokers: * *p* < 0.05; ventilatory threshold (VTH), and maximal exercise (Max).
